# Procyanidin capsules attenuate PI3K/AKT-mediated mitochondrial dysfunction and accelerate skin wound healing in diabetic mice

**DOI:** 10.1016/j.mtbio.2026.103029

**Published:** 2026-03-14

**Authors:** Yifan Ping, Jiaying Wang, Shaoyin Wei, Wen Tong, Wanhang Li, Junxin Ren, Siyi Wang, Xinyi Yao, Liyang Zheng, Zelin Cao, Dong Yang, Cuie Wen, Shengbin Huang, Shufan Zhao

**Affiliations:** a Institute of Stomatology, School and Hospital of Stomatology, Wenzhou Medical University, Wenzhou, China; b The Quzhou Affiliated Hospital of Wenzhou Medical University, Quzhou People's Hospital Department of Prosthodontics, School and Hospital of Stomatology, Wenzhou Medical University, Wenzhou, China; c Key Laboratory of Colloid and Interface Chemistry of the Ministry of Education, School of Chemistry and Chemical Engineering, Shandong University, Jinan, Shandong, 250100, China; d Department of Oral and Maxillofacial Surgery, School of Stomatology, Wenzhou Medical University, Wenzhou, China; e The International Peace Maternity and Child Health Hospital of China Welfare Institution, School of Medicine, Shanghai Jiao Tong University, Shanghai, China; f Shanghai Key Laboratory of Embryo Original Diseases, Shanghai Jiao Tong University, Shanghai, China; g School of Ophthalmology and Optometry, Eye Hospital, School of Biomedical Engineering, Wenzhou Medical University, Wenzhou, Zhejiang, 325035, China; h Wenzhou Institute, University of Chinese Academy of Sciences, Wenzhou, Zhejiang, 325001, China; i Centre for Additive Manufacturing, School of Engineering, RMIT University, Melbourne, Victoria, 3001, Australia

**Keywords:** Wound healing, Mitochondrial dysfunction, Oxidative stress, PI3K/AKT, Procyanidin capsules, Diabetes mellitus

## Abstract

Hyperglycemia-induced oxidative stress considerably hinders healing of diabetic wounds, primarily due to mitochondrial dysfunction. This pathology leads to an excessive production of reactive oxygen species (ROS), disrupts respiratory chain function, and impairs energy metabolism. This study introduces procyanidin (PC) capsules designed to target the phosphatidylinositol 3-kinase/protein kinase B (PI3K/AKT) signaling pathway. The PC capsules demonstrate a sustained ability to scavenge radicals, thereby directly lowering ROS levels and specifically activating the PI3K/AKT pathway. Through this activation, the capsules restore mitochondrial function by reducing mitochondrial reactive oxygen species, stabilizing mitochondrial membrane potential, and restoring energy production. Additionally, cell experiments reveal that the capsules significantly boost the migration of fibroblasts and enhance the angiogenic activity of endothelial cells, indicating the protective effects of PC on crucial cell functions involved in wound healing. In a chronic skin wound model of diabetic mice, the PC capsules are found to accelerate wound closure by promoting collagen deposition, suppressing excessive inflammation, and minimizing mitochondrial oxidative damage. Using the PI3K inhibitor LY294002, we further verified that the pro-migratory and pro-angiogenic effects of PC capsules are largely dependent on the PI3K/AKT signaling pathway. Our novel findings suggest that PC capsules stimulate PI3K/AKT-mediated repair of mitochondrial function, presenting a potential therapeutic approach for treating refractory diabetic wounds.

## Graphical abstract

**Figure:**
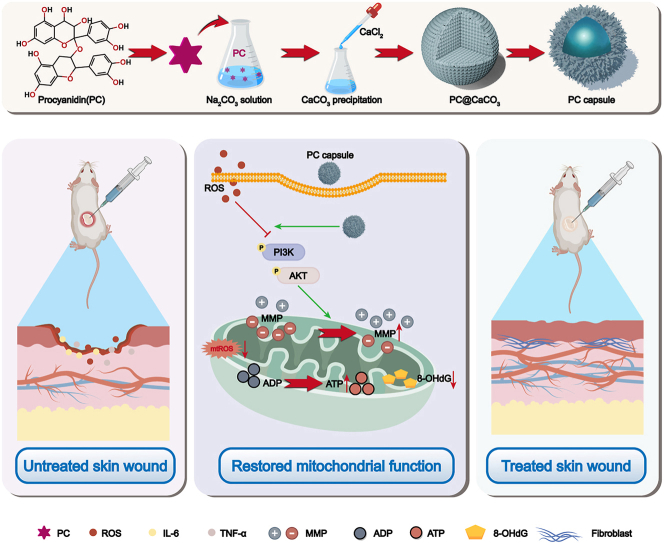

## 1 Introduction

Diabetic wounds, a severe and common complication of diabetes, are characterized by delayed healing and significantly impact patients' quality of life [1]. With the global rise in diabetes prevalence, diabetic foot ulcers develop in approximately one-quarter of patients, posing a significant burden on healthcare systems and socioeconomic resources [2,3]. The core pathological features of these wounds include a persistent inflammatory response, fibroblast dysfunction, and inadequate blood-vessel formation, all of which are associated with mitochondrial dysfunction caused by high blood sugar levels [4,5]. Mitochondria, which are crucial for regulating cellular energy and oxidative stress, can become overloaded in hyperglycemic conditions, resulting in excessive production of reactive oxygen species (ROS) [6]. This induces mitochondrial dysfunction characterized by elevated ROS levels, DNA damage, membrane potential collapse, and reduced adenosine triphosphate (ATP) synthesis, ultimately impairing essential wound-healing mechanisms such as inflammatory cell polarization, cellular migration, and angiogenesis [[7], [8], [9]]. Based on this understanding, the restoration of mitochondrial function, including energy-producing capacity and redox balance, has emerged as a promising therapeutic approach for promoting tissue regeneration in diabetic wounds.

Although existing wound dressings and hydrogels can partially improve the wound environment, there is a pressing clinical need for specific strategies that target mitochondrial oxidative damage. Activation of the phosphatidylinositol 3-kinase/protein kinase B (PI3K/AKT) signaling pathway begins with PI3K catalyzing a phosphorylation reaction, leading to a series of events that ultimately activate AKT [10]. Activated AKT plays a key role in maintaining mitochondrial health in diabetic wound repair cells by regulating processes such as cell death and metabolism [11,12]. Specifically, it can boost the expression of antioxidant enzymes that scavenge ROS, stabilize mitochondrial membrane potential (MMP), and restore energy-producing efficiency [[13], [14], [15]]. This enhances fibroblast migration and proliferation, promotes endothelial cell angiogenesis, and reduces chronic inflammation, thereby creating a favorable wound-healing environment [16,17]. However, in a hyperglycemic environment, certain factors inhibit this pathway, leading to oxidative stress that ultimately impedes tissue repair and angiogenesis [18].

Natural polyphenols, known for their antioxidant and anti-inflammatory properties with minimal side effects, have emerged as promising alternatives for the management of chronic wounds [19]. Procyanidin (PC) stands out because of its potent antioxidant, anti-inflammatory, and angiogenic activities [20], as well as the ability to inhibit cell death in various cell types [21]. Recent research demonstrated that PCs alleviated mitochondrial dysfunction induced by high glucose levels [22]. These compounds may activate the PI3K/AKT signaling pathway, which would explain their physiological effects. However, the clinical application of PCs for the treatment of chronic diabetic wounds is limited by their unstable nature and susceptibility to oxidative degradation [23]. Therefore, developing strategies to enhance their bioavailability is essential for fully exploiting their therapeutic benefits.

To overcome the inherent instability of PCs, which severely limits their clinical translation [24,25], we developed a novel formulation—PC capsules—via a self-polymerization process. Unlike conventional PC solutions that are prone to rapid oxidative degradation, these capsules are engineered to provide superior physicochemical stability and sustained antioxidant release. This formulation significantly enhances bioavailability and extends the duration of therapeutic action. The key innovative advantages of our PC capsules lie in their remarkable long-term antioxidant capacity and controlled release profile, which together ensure that effective drug concentrations are maintained in the challenging diabetic wound microenvironment. Consequently, these capsules exhibit low toxicity, excellent skin compatibility, and, most importantly, the ability to continuously scavenge ROS over extended periods. Transcriptome and Western blot experiments validated that these capsules activated the PI3K/AKT signaling pathway, protected mitochondria from oxidative damage, maintained MMP, restored mitochondrial function, and increased ATP production. Consequently, this enhanced fibroblast migration and endothelial cell angiogenesis promote diabetic wound healing. The PC capsules used in this study offer new therapeutic possibilities for diabetic wounds and chronic inflammatory diseases, particularly by regulating PI3K/AKT pathway–mediated mitochondrial homeostasis.

## 2 Material and methods

### 2.1 Scanning electron microscopy detection

The prepared capsules were re-dispersed in pure water and diluted to an appropriate concentration, and 10 μL was dropped onto a clean silicon wafer. After vacuum drying at 55 °C, the silicon wafer was adhered using the conductive adhesive onto the scanning electron microscope sample stage and sprayed with gold for 40 s, and the microstructure of the capsules was observed through scanning electron microscopy (SEM).

### 2.2 Transmission electron microscopy detection

The prepared PC capsules were re-dispersed in an aqueous solution and diluted to the appropriate concentration, and 5 μL was dropped onto a carbon support membrane. The capsules were dried in a vacuum oven for 2 h and their morphologies were observed using transmission electron microscopy.

### 2.3 Infrared spectroscopy and X-ray photoelectron spectroscopy detection

The prepared PC capsules were centrifuged at 4000 rpm for 4 min, the supernatant was removed, and they were vacuum dried at 55 °C overnight to obtain a powder. A portion of the powder was used for compression testing using infrared spectroscopy and another portion for X-ray photoelectron spectroscopy (XPS) detection to investigate the capsule formation process.

### 2.4 Ability of the capsules to remove H_2_O_2_

First PC capsules of different concentrations were mixed with hydrogen peroxide (H_2_O_2_) solution (60 μM), then 200 μL vitamin C (Vc) solution, PC solution, and PC capsules (0.25 mg/mL) were each mixed with equal volumes of H_2_O_2_ solution. The three solutions were shaken at 60 rpm for 5 h and then centrifuged at 10,000 rpm for 5 min. Fifty microliters of the supernatant and 950 μL of the FOX solution were quickly mixed into them and they were incubated at room temperature for 30 min. The absorbance of the mixed solutions was measured at 560 nm using a UV-visible spectrometer. After placing the PC solution and capsules in a non-light-shielded state at room temperature for 30 days, the above operation was repeated to explore the long-term ability of the three solutions to remove H_2_O_2_.

### 2.5 Ability of the capsules to remove superoxide anions

A total superoxide dismutase activity detection kit was used to determine the ability of the PC capsules to remove superoxide anions. After mixing different concentrations of the PC capsules with the working solution, the mix was incubated at 37 °C for 30 min and the absorbance was measured at 450 nm.

### 2.6 Ability of the capsules to scavenge DPPH free radicals

A 2,2-diphenyl-1-picrylhydrazyl (DPPH) radical scavenging assay kit was used to determine the ability of PC capsules to scavenge DPPH radicals. PC capsules of different concentrations were mixed with H_2_O_2_ solution (60 μM) or 200 μL of Vc solution, then the two PC solutions and PC capsules (0.25 mg/mL) were mixed with equal volumes of H_2_O_2_ solution and allowed to stand in the dark at room temperature for 30 min. The absorbance of the solutions was measured at 515 nm using an enzyme-linked immunosorbent assay (ELISA) reader. The Vc solution was removed by placing the PC solutions and PC capsules in a non-light-shielded state at room temperature for 30 Days, and the above operation was repeated to explore the long-term ability of the three solutions to scavenge DPPH free radicals.

### 2.7 Ability of the capsules to eliminate ABTS free radicals

We used an 2,2′-azino-bis (3-ethylbenzothiazoline-6-sulfonic acid (ABTS) free-radical scavenging assay kit to determine the ability of PC capsules to scavenge ABTS free radicals. PC capsules of different concentrations were mixed with equal volumes of H_2_O_2_ solution (60 μM) or 200 μL of Vc solution, then the two PC solutions and PC capsules (0.25 mg/mL) were mixed with equal volumes of H_2_O_2_ solution, thoroughly mixed, and allowed to stand at room temperature in the dark for 6 min. The absorbance of the samples was measured at 405 nm. After placing the PC solutions and capsules in a non-light-shielded state at room temperature for 30 days, the above operation was repeated to explore the long-term ability of the three solutions to scavenge ABTS free radicals.

### 2.8 Total antioxidant capacity of the capsules

A total antioxidant capacity assay kit was used to determine the total antioxidant capacity of the capsules. Vc, PC solution, and PC capsules (500 μg/mL) were mixed evenly with the working solution and incubated at 37 °C for 8 h, and the absorbance value at 593 nm was determined using an ELISA reader. The higher the value, the stronger the total antioxidant capacity of the material. After placing the PC solution and capsules in a non-light-shielded state at room temperature for 30 days, the above operation was repeated to explore the long-term total antioxidant capacity of the three solutions.

### 2.9 Cell culture and treatment

To evaluate the critical cellular processes impaired in diabetic wound healing, we selected two representative cell lines for *in vitro* assessment. Human umbilical vein endothelial cells (HUVECs) serve as a well-established model for studying angiogenesis and endothelial function, both of which are essential for vascular regeneration and are frequently compromised in diabetic wounds [26]. NIH/3T3 fibroblasts were employed to investigate fibroblast migration and proliferation, key events in wound contraction and extracellular matrix remodeling that are dysregulated under hyperglycemic conditions [27]. Human umbilical vein endothelial cells (HUVECs) (CRL-1730, USA, RRID: CVCL_2959) and NIH/3T3 cells (CRL-1658, USA, RRID: CVCL_0594) used in this study were purchased from American Type Culture Collection. All cells tested negative for mycoplasma and were regularly subjected to short tandem repeat (STR) profiling to confirm their identity. The cells were maintained in Dulbecco's Modified Eagle Medium (DMEM/Sodium Pyruvate [+]; C11995500BT, Gibco, USA) with 10% fetal bovine serum (04-001-1ACS, BI, Israel) and 1% antibiotics (100 IU/mL penicillin G and 100 μg/mL streptomycin; 15140-122, Gibco, USA) and streptomycin at 37 °C in a humidified atmosphere containing 5% CO_2_. The cells were incubated with or without H_2_O_2_ (Sigma), PC capsules, N-acetylcysteine (NAC) or LY294002 for various periods.

### 2.10 Cytotoxicity testing

For cytotoxicity assessment, cells were seeded in 96-well plates (1 × 10^4^ cells/well) and exposed to various concentrations of PC capsules (0.03–0.25 mg/mL) for 24 h. Cell viability was determined using 3-(4,5-dimethylthiazol-2-yl)-2,5 diphenyl tetrazolium bromide (MTT) assay (M2128, Sigma, Germany), with absorbance measured at 490 nm. Parallel live/dead staining was performed using calcein-AM/PI (40747ES80, Yeasen, China) on cells cultured in 48-well plates (1.5 × 10^4^ cells/well), with fluorescence imaging conducted using a Zeiss Axio Observer3 microscope.

### 2.11 Cell viability assay

Cells were inoculated at a density of 1 × 10^4^ cells/well in a 96-well plate. After 24 h, HUVECs and NIH/3T3 cells were stimulated with different concentrations of H_2_O_2_. After 6 h of cultivation, the complete culture medium was replaced with MTT and the optical density was measured according to the above method.

Based on the MTT results, the final working concentration of H_2_O_2_ in HUVECs and NIH/3T3 cells was 0.90 mM. Cells were inoculated at a density of 1 × 10^4^ cells/well in a 96-well plate. After 24 h, both types of cells were pretreated with either different concentrations of PC capsules for 12 h or NAC for 2 h. After co-culturing cells with PC capsules or NAC and H_2_O_2_ for 6 h, cell viability was measured using MTT assay.

### 2.12 Wound-healing migration assay

HUVECs and NIH/3T3 cells were plated in 12-well plates at a density of 1 × 10^5^/mL. After the formation of a monolayer, HUVECs were incubated in a serum-free medium for 12 h and a line was scratched using a sterilized 200 μL pipette tip. The scratch-wounded cells were pretreated with either PC capsules for 12 h or NAC for 2 h, followed by H_2_O_2_ stimulation for 24 h. Multiple non-overlapping fields along the scratch were captured at 0 h and 24 h post-scratch. Quantitative analysis of the wound closure area was performed using ImageJ software (version 1.53, National Institutes of Health, USA). For each field, the scratch area at 0 h (A_0_) and 24 h (A_24_) was manually traced using the "Freehand Selections" tool. The percentage of wound closure was calculated using the formula: [(A_0_ - A_24_)/A_0_] × 100%. The final value for each biological replicate represents the average percentage closure from at least three random microscopic fields. Threshold settings were not applied, as the cell-free scratch area was distinctly identifiable from the confluent cell monolayer.

### 2.13 Tube-formation assay

A tube-formation assay was conducted using growth factor-reduced Matrigel (354234; Corning, USA). Briefly, 96-well plates were coated with Matrigel and polymerized at 37 °C for 1 h. HUVECs (5 × 10^4^ cells/mL), harvested using trypsin/ethylenediaminetetraacetic acid, were seeded onto the polymerized matrix and incubated for 6 h at 37 °C. Tubular networks in each well were visualized under a phase-contrast microscope ( × 20 magnification). For quantification, three non-overlapping fields per well were captured. The angiogenic parameters—total tube length, number of branch points (branch sites), and number of tube nodes—were analyzed using the “Angiogenesis Analyzer” plugin for ImageJ software. Briefly, images were converted to 8-bit, and a consistent threshold was applied to create a binary image highlighting the tubular structures. The plugin's default settings for skeletonization and network analysis were used. The total tube length (sum of the length of all segments in pixels, converted to micrometers using the image scale), the number of branch points (junctions where three or more segments meet), and the number of tube nodes (the total number of junctions and endpoints in the network) were automatically calculated by the plugin. Data from three independent wells were pooled for each experimental condition.

### 2.14 Mitochondrial ROS assay

Mitochondrial ROS levels were assessed using the MitoSOX Red reagent (M36008; Invitrogen, 5 μM). Following treatment, HUVECs and NIH/3T3 cells were stained for 30 min at 37 °C, with fluorescence imaging performed at 510/580 nm excitation/emission. Quantitative analysis was conducted by measuring the mean fluorescence intensity using ImageJ software.

### 2.15 Measurement of MMP

HUVECs and NIH/3T3 cells (1 × 10^4^ cells/mL) were pretreated with either PC capsules for 12 h or NAC for 2 h and then stimulated with H_2_O_2_ for 6 h. The MMP was assessed using tetramethylrhodamine, methyl ester, perchlorate (TMRM) (T668, Invitrogen, USA, 10 nM). Following treatment, HUVECs and NIH/3T3 cells were stained for 30 min at 37 °C, washed thrice with phosphate-buffered saline (PBS), and immediately imaged using fluorescence microscopy. Quantitative analysis was performed using Image-Pro Plus 6.0, with MMP expressed as the ratio of red to green fluorescence intensity.

### 2.16 ATP synthesis assay

Cellular ATP levels were quantified using a commercial ATP assay kit (A22066; Invitrogen). Cells were lysed in the kit-provided buffer, followed by centrifugation (12,000 rpm, 4 °C, 5 min). Supernatants were analyzed using a microplate reader (M5, MD, USA) with 100 μL ATP detection buffer. ATP concentrations were determined against a standard curve (1 nM–1 μM) following established protocols.

### 2.17 In vivo biocompatibility of capsules

C57BL/6J mice (8–10 weeks old) were purchased from WeiTonglihua Biotechnology (Beijing, China). All animal experiments were performed in accordance with the NIH Guide for the Care and Use of Laboratory Animals (NIH Publication No. 80-23, revised in 1978) and were approved by the Committee on Experimental Animal Welfare and Ethics of the Wenzhou Institute, University of Chinese Academy of Sciences (No. WIUCAS25071704).To evaluate the biocompatibility of PC capsules, C57BL/6J mice were randomly assigned to experimental groups (n = 6–8 mice per group) and received subcutaneous administration of PC capsules (100 μL). PBS-injected mice served as controls. After 7 days, serum biochemical parameters, including alanine aminotransferase (ALT) and aspartate aminotransferase (AST), as well as renal function parameters, including blood urea nitrogen (BUN) and creatinine (CRE), were analyzed. This was followed by histological examination of major organs (heart, liver, spleen, lungs, and kidneys) using hematoxylin and eosin (H&E) staining. All biochemical analyses and histological evaluations were performed under blinded conditions, with investigators unaware of group allocation during data acquisition and analysis to minimize experimental bias.

### 2.18 Immunohistochemical staining

Wound tissues were fixed in 4% paraformaldehyde, paraffin-embedded, and sectioned at 5 μm. Sections were deparaffinized, rehydrated, and stained with H&E or Masson's trichrome (Solarbio, China) following standard protocols.

For IHC, antigen retrieval was performed in sodium citrate buffer (pH 6.0) using microwave heating. After blocking with 10% normal goat serum, sections were incubated overnight at 4 °C with primary antibodies diluted in PBS with 1% BSA. The following antibodies were used:

Anti-phospho-AKT (#4060, CST, USA; 1:200)

Anti-phospho-PI3K (#AF3241, Affinity, China; 1:300)

Anti-IL-6 (ab233706, Abcam, UK; 1:250)

Anti-TNF-α (60291-1-Ig, Proteintech, China; 1:200)

Sections were then incubated with HRP-conjugated secondary antibodies (ZSGB-Bio, China) for 1 h at 37 °C, developed with DAB substrate (ZSGB-Bio, China), and counterstained with hematoxylin. All slides were scanned digitally using a Pannoramic MIDI scanner (3DHISTECH, Hungary).

### 2.19 Chronic wound healing

The therapeutic efficacy of the PC capsules was evaluated in a streptozotocin-induced diabetic wound model. Male C57BL/6 mice (8–10 weeks old, weighing 18–21 g) were used in this study to avoid potential confounding effects of hormonal cycles on wound healing. Mice were housed under standard conditions with a 12 h light/dark cycle and free access to food and water. Animals were randomly assigned to different experimental groups, with 6–8 mice per group. All wound treatments and outcome assessments were conducted under a randomization and blinding protocol. Investigators responsible for wound area measurement and histological analyses were blinded to the group allocation throughout data collection and analysis to minimize experimental bias. Diabetes was induced by intraperitoneal injections of streptozotocin (STZ, 50 mg/kg/day in citrate buffer, pH 4.5) for five consecutive days [28,29]. Non-fasting blood glucose levels were monitored weekly from the tail vein using a glucometer. Mice with sustained blood glucose levels >13 mmol/L for two consecutive weeks were considered diabetic and included in the study. One week after the confirmation of stable hyperglycemia, the mice were anesthetized, and the dorsal hair was shaved and depilated. Full-thickness excisional wounds were created on the midline of the back using a sterile 8 mm diameter biopsy punch. Wounds with irregular shapes or significant size deviations (>10% from the mean) were excluded. The mice were then randomly assigned to experimental groups. Wound closure was monitored on days 1, 7, and 14 by capturing standardized digital photographs with a reference scale. The wound area was quantified using ImageJ software, and the percentage of wound closure was calculated relative to the initial wound area (day 0). Tissues were harvested at the respective endpoints for histological (H&E, Masson's trichrome) and IHC (p-PI3K, p-AKT, IL-6, TNF-α) analysis. Oxidative stress was assessed using 8-OHdG immunofluorescence.

### 2.20 RNA-seq and bioinformatics analysis

Total RNA was extracted from wound tissues (untreated and PC capsules–treated groups, n = 3 each) using TRIzol reagent (Invitrogen). RNA quality was verified (RIN ≥7.0), and libraries were prepared with the NEBNext Ultra II kit. Paired-end sequencing (150 bp) was performed on an Illumina NovaSeq 6000 platform (Shanghai Personal Biotechnology Co.). Raw reads were processed with Fastp, aligned to the mouse genome (GRCm39) using STAR, and gene expression quantified with StringTie. Differentially expressed genes (DEGs) were identified with DESeq2 using thresholds of |log_2_FC| > 0.585 (fold change >1.5) and adjusted p-value <0.05 (Benjamini–Hochberg correction). Functional enrichment analysis of DEGs was performed using the Gene Ontology (GO) and Genes and Genomes (KEGG) pathway databases (p < 0.05, hypergeometric test).

### 2.21 Western blotting assay

Protein extracts from HUVECs and NIH/3T3 cells were prepared using a lysis buffer (#9803, Cell Signaling Technology, USA) and quantified using a Bradford assay (#23236, Thermo Fisher Scientific, USA). Equal amounts of protein were separated by sodium dodecyl sulfate–polyacrylamide gel electrophoresis and transferred to polyvinylidene fluoride membranes (Bio-Rad, Hercules, CA, USA). After blocking (5% skim milk/bovine serum albumin, 1.5 h), the membranes were incubated overnight at 4 °C with the primary antibodies against p-AKT (#4060, CST, USA; 1:1000), p-PI3K (#AF3241, Affinity, China; 1:1000), total AKT (#2920, CST, USA; 1:1000), and PI3K (#4249, CST, USA; 1:1000). Horseradish peroxidase–conjugated secondary antibodies were applied (1.5 h, room temperature), followed by chemiluminescence detection (#1251473, Thermo Fisher Scientific, USA). The bands were quantified using ImageJ (v1.80) after imaging (Bio-Rad ChemiDoc Touch v5.1).

### 2.22 Statistical analysis

All data are presented as mean ± standard deviation (SD) of triplicate experiments. Statistical analysis was performed using one-way analysis of variance, followed by Tukey's post-hoc test using GraphPad Prism 8.0 software (GraphPad Prism Software Inc., San Diego, CA, USA). Statistical significance was confirmed when the p-value was <0.05.

## 3 Results

### 3.1 Preparation and characterization of the PC capsules

(Fig. 1A) shows the preparation process of PC capsules. This process involved the self-aggregation of high-concentration PC onto calcium carbonate particles, followed by removal of the calcium carbonate using hydrochloric acid, leaving behind PC capsules encapsulated on the outer surface of the calcium carbonate. First, the surface morphology, size, and characteristics of PC@CaCO_3_ and PC capsules were observed using SEM (Fig. 1B and C), with PC@CaCO3 and PC capsules measuring approximately 2–5 μm in size. After nuclear removal, the PC capsules exhibited hollow structures. Inverted microscopy and laser scanning confocal microscopy (CLSM) confirmed that the PC capsules had a capsule-like structure enclosed by a thin wall (Fig. 1D and E). In addition, the permeability of the PC capsules was tested using fluorescein isothiocyanate (FITC)-dextran of different molecular weights (Fig. 1F and G). CLSM images show the interaction of the PC capsules with FITC-dextran at 40 kDa and 500 kDa. The PC capsules had a porous structure and holes sized >40 kDa. Fourier transform infrared (FTIR) spectroscopy confirmed that the PC capsules had the same characteristic peaks as PC, indicating that the PC capsules were composed of PC (Fig. 1H). In addition, the absorption peaks shifted toward lower frequencies at 3391 cm^−1^ and 1720 cm^−1^, and the vibrational bands widened, indicating that the PC capsules were mainly formed by hydrogen-bonding interactions. Furthermore, XPS, used to analyze the elemental composition of the PC capsules, indicated that calcium (Ca) accounted for only 0.76% (Fig. 1I and J). Thus, the synthesized PC capsules were mainly composed of carbon (C) and oxygen (O) elements, assembled through hydrogen bonding between PC molecules using calcium carbonate as a template.

**Fig. 1:**
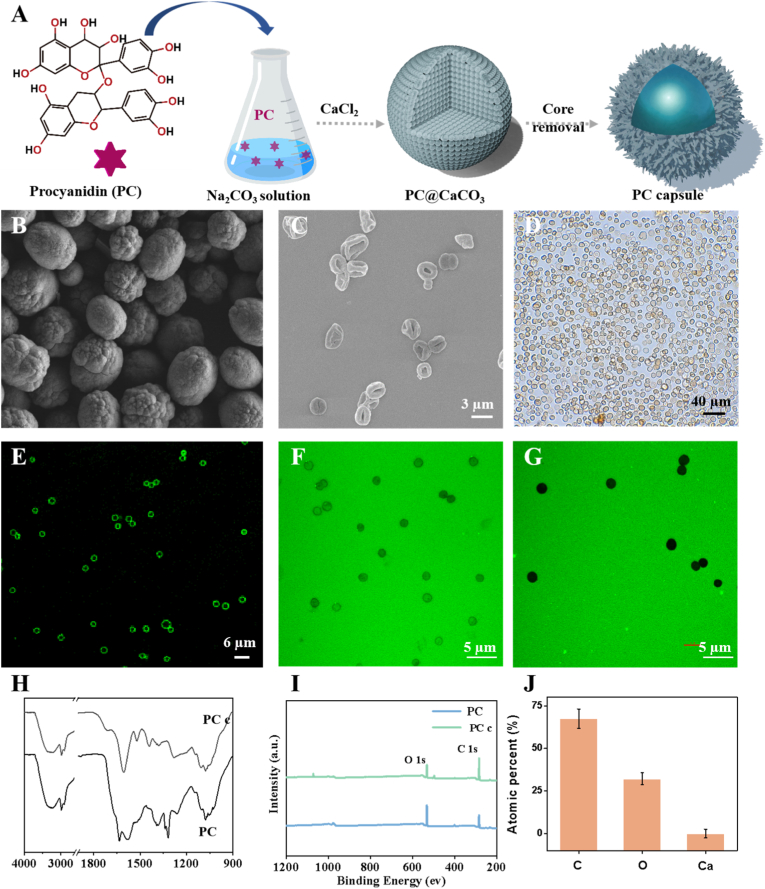
(A) Preparation of PC capsules; (B) SEM of PC@CaCO3; (C) SEM; (D) inverted microscopy and (E) CLSM of capsules doped with PC capsules. CLSM images of PC capsules against FITC-dextran with Mw of: (F) 40 kDa and (G) 500 kDa. (H) FTIR spectra of PC and PC capsules; (I) XPS spectra of PC capsules; (J) element percentages of PC capsules from XPS data in (I).

### 3.2 In vitro antioxidant capacity of the PC capsules

**Fig. 2:**
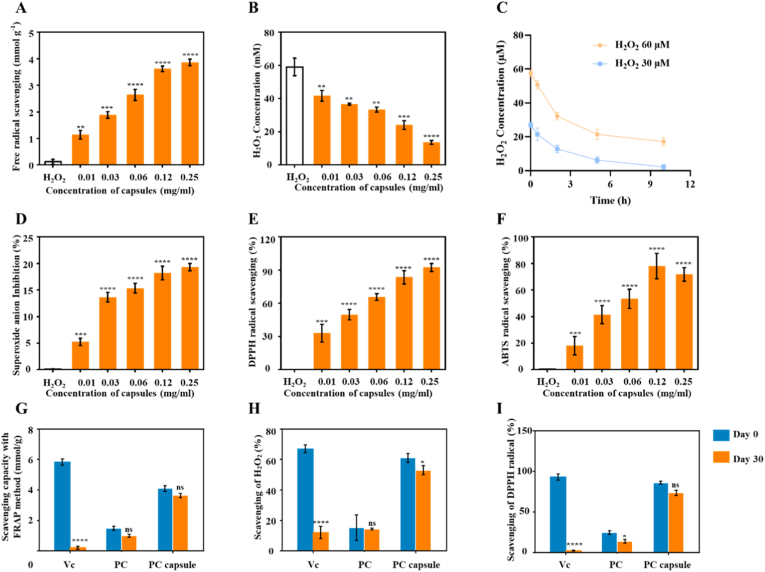
ROS scavenging and RNS scavenging ability of PC capsules: (A) free-radical scavenging ability and (B) H_2_O_2_ scavenging ability of different concentration of PC capsules; (C) ability of PC capsules to clear different concentrations of H_2_O_2_ over time; (D) O_2_-scavenging ability; (E) DPPH and (F) ABTS radical scavenging ability of different concentrations of PC capsules; (G) total antioxidant ability; (H) H_2_O_2_ scavenging ability and (I) DPPH radical scavenging ability of Vc, PC, and PC capsules. Data on the left of Vc, PC, and PC capsules in (G), (H), and (I) represent the short-term antioxidant effects and on the right represent the long-term (30 days) scavenging effects. Data are presented as mean ± standard deviation (SD) with n = 3 independent biological replicates per group. Statistical analysis was performed using one-way ANOVA followed by Tukey's post-hoc test. Significance markers are defined as: ∗∗p < 0.01, ∗∗∗p < 0.001, ∗∗∗∗p < 0.0001.

This study investigated the scavenging effects of different concentrations of PC capsules on various free radicals and the capsules’ antioxidant capacities. Fig. 2A shows that the PC capsules exhibited significant free-radical scavenging ability at all test concentrations and their scavenging effect appeared more prominent at high concentrations. Specifically, the scavenging ability of PC capsules toward (H_2_O_2_) (Fig. 2B and C), superoxide anion (O^2−^), DPPH, and ABTS radicals (Fig. 2D, E, F) increased with increasing concentration, reflecting their efficient antioxidant properties.

As shown in Fig. 2, the ability of the PC capsules to scavenge H_2_O_2_ and DPPH was 3.14, 2.77, and 3.99 times, respectively, that of the PC solution. The scavenging ability of the PC capsules toward H_2_O_2_ and DPPH was comparable to that of Vc. The Vc solution, PC solution, and PC capsules were stored at room temperature for 2 months, and the above method was used to determine their long-term ability to scavenge ROS and reactive nitrogen species (RNS). The results showed that after 2 months of storage, the total antioxidant, DPPH scavenging, and H_2_O_2_ scavenging capacities of the Vc solution decreased by 96.56%, 97.96%, and 92.21%, respectively, whereas the antioxidant capacity of the PC solution and PC capsules decreased by only up to 22% (Fig. 2G, H, I).

### 3.3 In vitro biocompatibility of the PC capsules

**Fig. 3:**
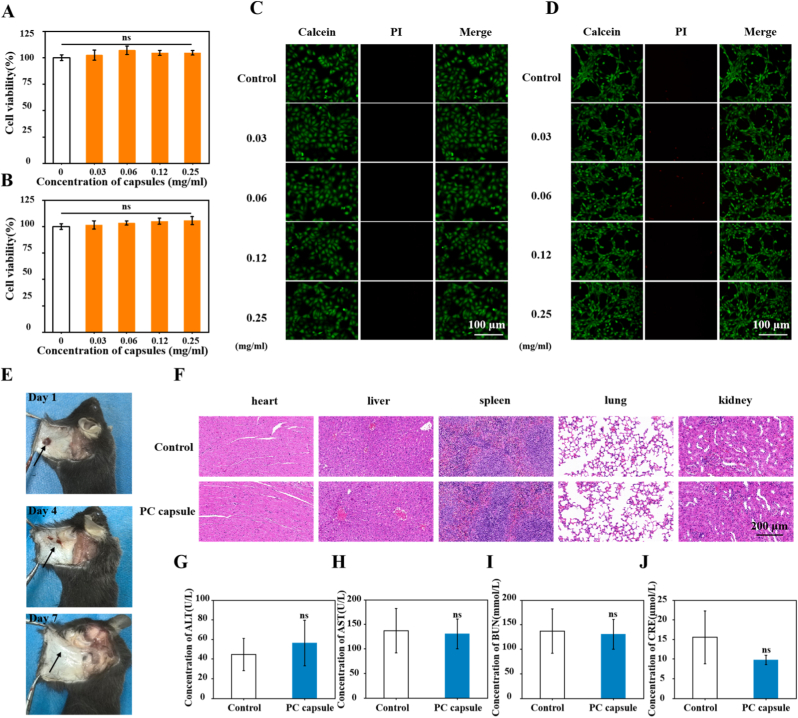
Biocompatibility of PC capsules: (A–B) cell viability of HUVECs (A) and NIH/3T3 cells (B) treated with different concentrations of PC capsules for 24 h detected by MTT assay; (C–D) toxic staining of PC capsules on HUVECs and NIH/3T3 cells detected by living/dead cell staining; (E) photos of the backs of mice after receiving a subcutaneous injection of PC capsules and subcutaneous PC capsules after injection at day 4 and day 7; (F) slices of major organs (heart, liver, spleen, lungs, and kidneys) in mice after 14 days of PC capsule administration; scale bar = 100 μm. Serum liver function indicators were: (G) ALT and (H) AST. Serum renal function indicators were: (I) BUN and (J) CRE. Data are presented as mean ± standard deviation (SD) with n = 3 independent biological replicates per group. Statistical analysis was performed using one-way ANOVA followed by Tukey's post-hoc test. Significance markers are defined as: ns (p > 0.05).

PC capsules need to have excellent biocompatibility to qualify as promising candidates for diabetic wound healing. Cytotoxicity was evaluated by treating HUVECs and NIH/3T3 cells with different doses of the PC capsules for 24 h and then measuring cell viability. The viability of HUVECs treated with different concentrations of the PC capsules exceeded 100% and no statistical differences were observed between the groups (Fig. 3A). Similarly, in NIH/3T3 cells, even at the highest concentration of the PC capsules, cell viability remained above 100% and no significant differences were observed between the treatment and control groups (Fig. 3B). This indicated that the PC capsules had no adverse effects on the cells. To further determine the effect of the PC capsules on cells, live/dead staining of HUVECs and NIH/3T3 cells was performed under identical conditions. Under treatment with different doses of the PC capsules, no significant dead cell staining was observed in any group (Fig. 3C and D), which further indicated the low cytotoxicity of the PC capsules. The data showed that the PC capsules had good biocompatibility, achieving a good balance between overall performance and cell compatibility.

To assess the biosafety and degradation profile of the PC capsules in vivo, approximately 100 μL of the PC capsule suspension was administered via subcutaneous injection to mice. The injection sites were carefully examined at predetermined time points (0, 4, and 7 days post-administration). After euthanasia, the skin tissue surrounding the injection site was dissected and subjected to macroscopic observation and photographic documentation. Notably, the residual PC capsules were partially detectable on day 4, whereas substantial degradation was observed on day 7, demonstrating the time-dependent biodegradation characteristics of the material (Fig. 3E).

Histopathological analysis revealed no significant morphological differences or pathological lesions in the major organs (heart, liver, spleen, lungs, and kidneys) between the experimental and control groups (Fig. 3F). Serum biochemistry showed comparable levels of hepatic markers (ALT and AST) and renal function parameters (BUN and CRE) between PC capsule–treated and control mice (Fig. 3G–J), demonstrating no apparent hepatotoxicity or nephrotoxicity. These results, combined with the long-term stability and degradability of the material, confirmed the excellent biosafety and tissue-repair potential of the PC capsules.

### 3.4 PC capsules increased cell viability in H_2_O_2_-stimulated HUVECs and NIH/3T3 cells

**Fig. 4:**
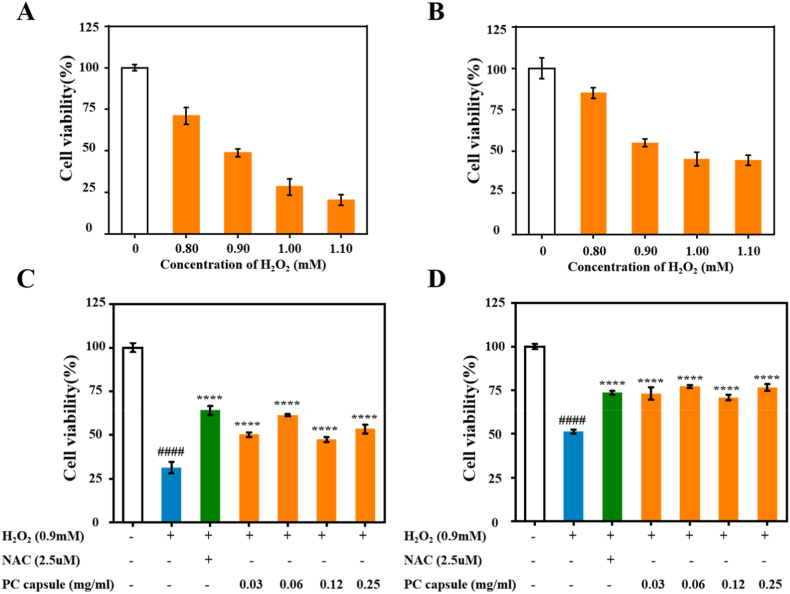
PC capsules increased cell viability under H_2_O_2_ stimulation: cell viability of HUVECs (A) and NIH/3T3 (B) cells treated with different concentrations of H_2_O_2_ for 6 h detected by MTT assay; viability of H_2_O_2_‐treated HUVECs (C) and NIH/3T3 cells (D) with different concentrations of PC capsule pretreatment. Data are presented as mean ± standard deviation (SD) with n = 3 independent biological replicates per group. Statistical analysis was performed using one-way ANOVA followed by Tukey's post-hoc test. Significance markers are defined as: ∗∗∗∗p < 0.0001.

After 6 h of treatment with different doses of H_2_O_2_, cell viability was detected by MTT assay. H_2_O_2_ treatment decreased cell viability in a dose-dependent manner. As H_2_O_2_ concentration increased, the cell survival rate decreased. Exposure to 0.90 mM H_2_O_2_ reduced the viability of HUVECs and NIH/3T3 cells to 48.79 ± 2.40% and 55.15 ± 2.45%, respectively, approximating the half-maximal inhibitory concentration (Fig. 4A and B); hence, as in previous studies [30,31], we used 0.90 mM H_2_O_2_ in subsequent experiments for these cell lines.

NAC, a mitochondria-targeting antioxidant, is the most widely used antioxidant in experimental cells, animal biology, and clinical studies [32]. In the HUVECs, NAC significantly reduced H_2_O_2_-induced damage and increased cell viability to 64.02 ± 2.43%. Similarly, we observed that different concentrations of the PC capsules significantly reduced H_2_O_2_-induced cell damage; the effect was most pronounced at a concentration of 0.25 mg/mL, with cell viability increasing to 61.39 ± 0.58%, similar to that observed with NAC treatment, whereas the cell viability after H_2_O_2_ treatment was only 31.37 ± 3.26% (Fig. 4C). In the NIH/3T3 cells, the cell viability increased to 77.04 ± 0.87% with 0.25 mg/mL of PC capsules. Compared to NAC, PC capsules were more effective in reducing H_2_O_2_ damage to cells. Therefore, we chose a concentration of 0.25 mg/mL for subsequent experiments (Fig. 4D). These results indicated that the PC capsules protected HUVECs and NIH/3T3 cells from H_2_O_2_-induced damage.

### 3.5 PC capsules restored cell function in H_2_O_2_-treated HUVECs and NIH/3T3 cells

**Fig. 5:**
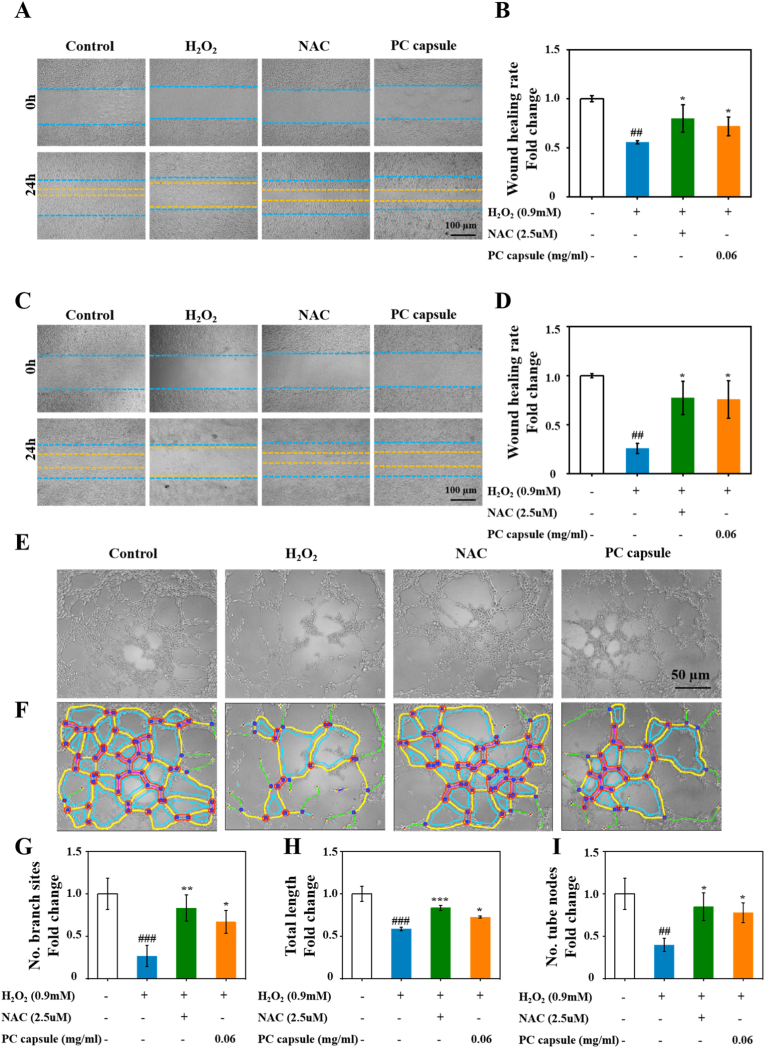
PC capsules restored cell function in H_2_O_2_-treated HUVECs and NIH/3T3 cells. HUVECs and NIH/3T3 cells were treated with PC capsules for 24 h and H_2_O_2_ for 6 h. (A) Images of HUVEC migration exposure to H_2_O_2_, NAC, PC capsules after 24 h; (B) quantification of percentage wound area remaining for HUVECs; (C) images of NIH/3T3 cell migration exposure to H_2_O_2_, NAC, PC capsules after 24 h; (D) quantification of percentage wound area remaining for NIH/3T3 cells; (E–F) digital images of endothelial cell microtubule formation after treatment with H_2_O_2_, NAC, and PC capsules. Quantification of (G) number of branch sites, (H) total lengths and (I) numbers of tube nodes. Data are presented as mean ± standard deviation (SD) with n = 3 independent biological replicates per group. Statistical analysis was performed using one-way ANOVA followed by Tukey's post-hoc test. Significance markers are defined as: ##p < 0.01, ###p < 0.001 (vs. control group); ∗p < 0.05, ∗∗p < 0.01, ∗∗∗p < 0.001 (vs. H_2_O_2_ group).

To assess the therapeutic potential of the PC capsules for cellular function, we performed a series of functional assays using H_2_O_2_-treated HUVECs and NIH/3T3 cells. The initial evaluation involved a scratch-wound healing assay to examine the PC capsule–mediated cell-migration capacity. As shown in Fig. 5A and C, 24 h after creating a scratch H_2_O_2_ treatment significantly reduced the migration rates of HUVECs and NIH/3T3 cells, whereas NAC and PC capsules reversed this effect and maintained good cell-migration rates under oxidative stress. In the HUVECs, the 24 h wound-healing rates in the H_2_O_2_, NAC, and PC capsule groups were 0.55 ± 0.01, 0.79 ± 0.13, and 0.71 ± 0.09, respectively, with no significant difference between the NAC and PC capsule groups (Fig. 5A and B); in the NIH/3T3 cells, the healing rates were 0.25 ± 0.05, 0.77 ± 0.16, 0.75 ± 0.19, respectively, with no significant difference between the NAC and PC capsule groups (Fig. 5C and D).

Lumen formation or tubulogenesis is an important step in angiogenesis. To evaluate the tubulogenic capability of PC capsule–treated HUVECs, we conducted a tubulogenesis assay using Matrigel. HUVECs exposed to either PC capsules or NAC were seeded onto Matrigel to examine their effects on tube formation. As illustrated in Fig. 5E–F, H_2_O_2_ significantly reduced neovascularization in the HUVECs. Cells exposed to PC capsules or NAC formed greater numbers of branch sites (Fig. 5G) and had greater total lengths (Fig. 5H) and numbers of tube nodes (Fig. 5I) than cells exposed to H_2_O_2_ (p < 0.05).

### 3.6 PC capsules alleviated H_2_O_2_-induced mitochondrial dysfunction

**Fig. 6:**
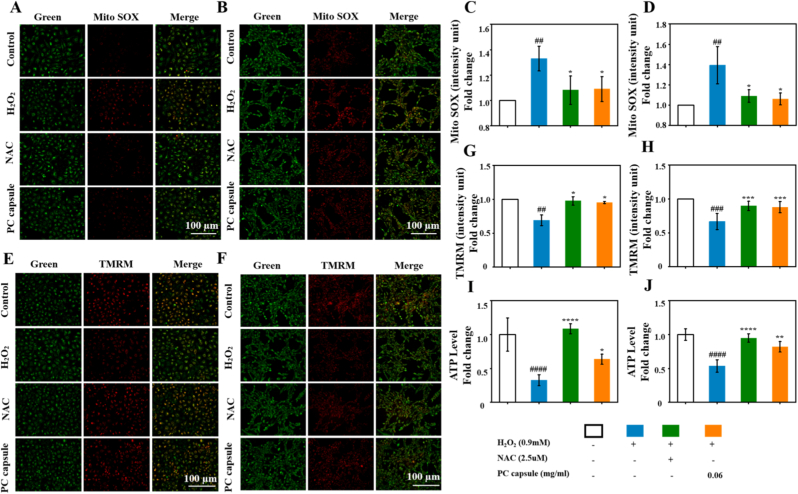
PC capsules improved mitochondrial function in H_2_O_2_-treated HUVECs and NIH/3T3 cells: (A–D) elimination of ROS from HUVECs (A, C) and NIH/3T3 (B, D) cells by PC capsules as determined by MitoSOX; (E–H) assessment of PC-mediated HUVECs (E, G) and NIH/3T3 cells (F, H) MMP using TMRM assays; (I–J) effects of PC capsules on ATP production in HUVECs (I) and NIH/3T3 cells (J). Data are presented as mean ± standard deviation (SD) with n = 3 independent biological replicates per group. Statistical analysis was performed using one-way ANOVA followed by Tukey's post-hoc test. Significance markers are defined as: ##p < 0.01, ###p < 0.001, ####p < 0.0001 (vs. control group); ∗p < 0.05, ∗∗p < 0.01, ∗∗∗p < 0.001, ∗∗∗∗p < 0.0001 (vs. H_2_O_2_ group).

The ischemia-reperfusion cycle during wound healing exacerbates oxidative stress, leading to mitochondrial impairment in HUVECs and NIH/3T3 cells. We examined whether the PC capsules could ameliorate these H_2_O_2_-mediated mitochondrial defects in both cell types. Mitochondrial ROS production was measured using MitoSOX Red staining. In the HUVECs (Fig. 6A–C) and NIH/3T3 cells (Fig. 6B–D), mitochondrial ROS levels increased 1.33 ± 0.09-fold (p < 0.01) and 1.39 ± 0.18-fold (p < 0.01), respectively, in the H_2_O_2_-treated group compared with the untreated control.

We assessed the MMP using tetramethylrhodamine methyl ester fluorescence staining. H_2_O_2_-stimulated HUVECs (Fig. 6E–G) and NIH/3T3 cells (Fig. 6F–H) exhibited significantly lower levels of MMP than the untreated control. Furthermore, H_2_O_2_ treatment inhibited ATP generation in both cell types (Fig. 6I and J) compared to untreated cells. These effects were restored to near-physiological levels by treatment with PC capsules or NAC. Notably, although NAC had a greater effect on improving ATP levels in the HUVECs than did the PC capsules, no difference was observed between the two in the NIH/3T3 cells. Moreover, no significant difference was observed in the recovery effects between PC capsules and NAC on mitochondrial ROS and MMP in the HUVECs and NIH/3T3 cells.

### 3.7 PC capsules attenuated mitochondrial dysfunction and accelerated skin wound healing in diabetic mice

**Fig. 7:**
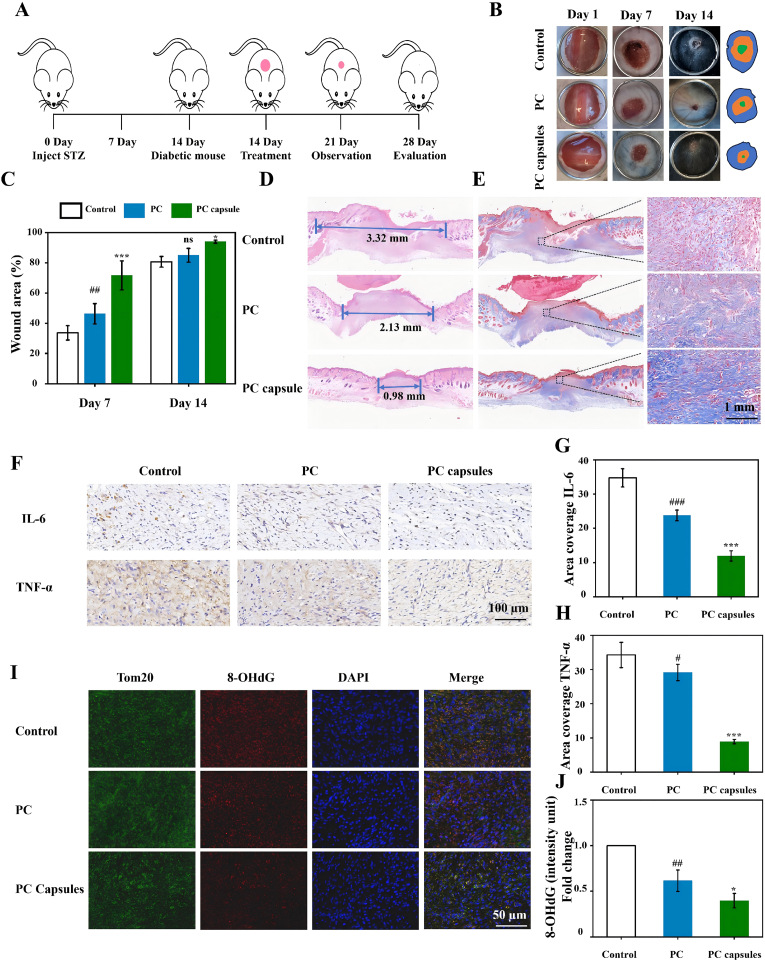
PC promoted wound healing in mice: (A) experimental design and timeline of interventions; (B) representative digital images of wound closure for control group, PC group, and PC capsule group on days 1, 7, and 14; (C) wound-closure rates in control group, PC group, and PC capsule group; (D) H&E staining of wound area on day 14; (E) Masson's staining of wound area on day 14 and partially enlarged Masson images; (F) expression of IL-6 and TNF-α as measured by IHC staining; (G–H) quantification of integral absorbance of IL-6 and TNF-α in IHC; (I) expression of 8-OHdG as measured by immunofluorescence; (J) quantitative analysis of 8-OHdG fluorescence intensity. In all experiments, data were presented as mean ± SD for biological replicates. #p < 0.05, ##p < 0.01, ###p < 0.001 (vs control group); ∗p < 0.05, ∗∗∗p < 0.001, (vs H_2_O_2_ group).

The therapeutic efficacy of the PC capsules was evaluated using a murine chronic-wound model. Fig. 7A illustrates the treatment timeline and Fig. 7B shows the wound appearance after 7- and 14-day treatments with either PC or PC capsules. Quantitative analysis (Fig. 7B–D) revealed progressive wound contraction from days 0 to 14, with the PC capsules showing superior healing capacity to those of PC alone and controls. Histological assessment (Fig. 7D) indicated enhanced epithelial regeneration in the PC capsule group. Masson's trichrome staining (Fig. 7E) confirmed significantly greater collagen deposition (intense blue staining) in the PC capsule–treated wounds versus other groups. IHC analysis of wound tissues revealed a progressive reduction in inflammatory cytokine expression, interleukin (IL)-6, and tumor necrosis factor-alpha (TNF-α) across control, PC, and PC capsule–treated groups (Fig. 7F–H). The suppression of pro-inflammatory markers demonstrated the superior anti-inflammatory efficacy of the PC capsules in hyperglycemic wound environments. In addition, immunofluorescence and quantitative analysis showed that compared with the control and PC groups, the expression of 8-hydroxy-2′-deoxyguanosine (8-OHdG), a biomarker of oxidative DNA damage, was significantly reduced in the PC capsule group. These results indicated that the wound-healing ability of the PC capsules was mainly due to their active and long-lasting antioxidant activity, which effectively reduced inflammation, alleviated mitochondrial dysfunction, and promoted collagen deposition (Fig. 7I and J).

### 3.8 PC capsule–mediated diabetic skin wound healing occurred via activation of PI3K/AKT signaling pathway

**Fig. 8:**
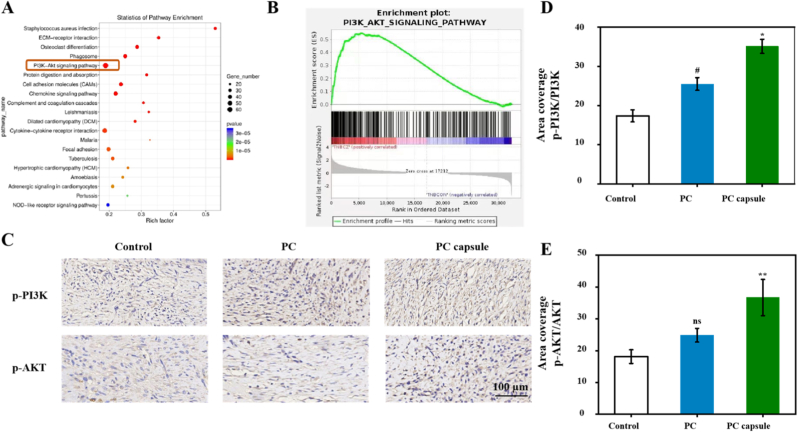
PC capsules promoted diabetes skin wound healing via activating PI3K/AKT signaling pathway: (A) top 20 KEGG pathway (signal transduction) analysis of differential genes between control group and PC capsule treatment group; (B) gene set enrichment analysis of regulated gene pathways with KEGG and Genomes databases; (C–E) IHC results showing increase in expression of p-PI3K in PC capsule group. Data are presented as mean ± standard deviation (SD) with n = 3 independent biological replicates per group. Statistical analysis was performed using one-way ANOVA followed by Tukey's post-hoc test. Significance markers are defined as: #p < 0.05 (vs control group); ∗p < 0.05, ∗∗p < 0.01 (vs PC group).

To analyze the potential mechanism of action of the PC capsules in the treatment of skin wounds in diabetic mice, we conducted RNA sequencing. Among the first 20 pathways detected by KEGG difference analysis, compared with the untreated group the PI3K/AKT signaling pathway was significantly activated after the PC capsules were used to treat chronic wounds in diabetic mice (Fig. 8A and B). To further characterize the transcriptional alterations within this pathway, we analyzed the top 10 differentially expressed genes associated with the PI3K/AKT signaling cascade. All selected genes were upregulated in the PC capsule–treated group. The expression patterns of these genes are presented as a heatmap in Supplementary Fig. 1. In addition, RT-qPCR validation was performed for four representative genes in HUVECs and NIH/3T3 cells. Consistent with the RNA sequencing results, all four genes exhibited significant upregulation following PC capsule treatment in both cell types. The quantitative results are shown in Supplementary Fig. 1. We further conducted relevant in vivo validation assays. IHC analysis showed that after treatment with the PC capsules, the expression levels of phosphorylated PI3K (p-PI3K) and phosphorylated AKT (p-AKT) increased (Fig. 8C, D, E). Our results suggest that the activated PI3K/AKT pathway plays a role in PC capsule–mediated skin wound healing in diabetes.

### 3.9 PC capsule–mediated cytoprotection via activation of PI3K/AKT signaling pathway

**Fig. 9:**
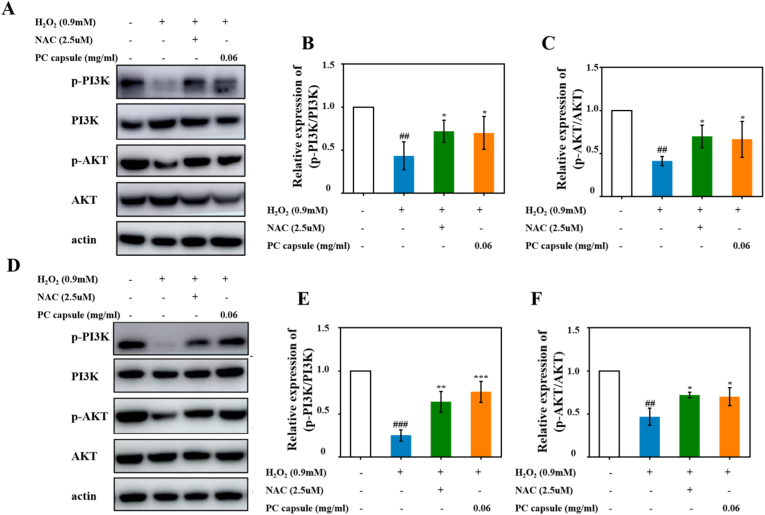
PC capsule–mediated cytoprotection via promoting activation of PI3K/AKT signaling pathway: expression of p-PI3K, PI3K, p-AKT, and AKT in HUVECs treated with PC capsules for 24 h and presence/absence of H_2_O_2_ for 6 h (A); and analysis of optical density values of p-PI3K/PI3K (B) and p-AKT/AKT (C); expression of p-PI3K, PI3K, p-AKT, and AKT in NIH/3T3 cells treated with PC capsules for 24 h and presence/absence of H_2_O_2_ for 6 h (D); and analysis of optical density values of p-PI3K/PI3K (E) and p-AKT/AKT (F). Data are presented as mean ± standard deviation (SD) with n = 3 independent biological replicates per group. Statistical analysis was performed using one-way ANOVA followed by Tukey's post-hoc test. Significance markers are defined as: #p < 0.05, ##p < 0.01, ###p < 0.001 (vs control group); ∗p < 0.05, ∗∗p < 0.01, ∗∗∗p < 0.001 (vs PC group or vs H_2_O_2_ group).

**Fig. 10:**
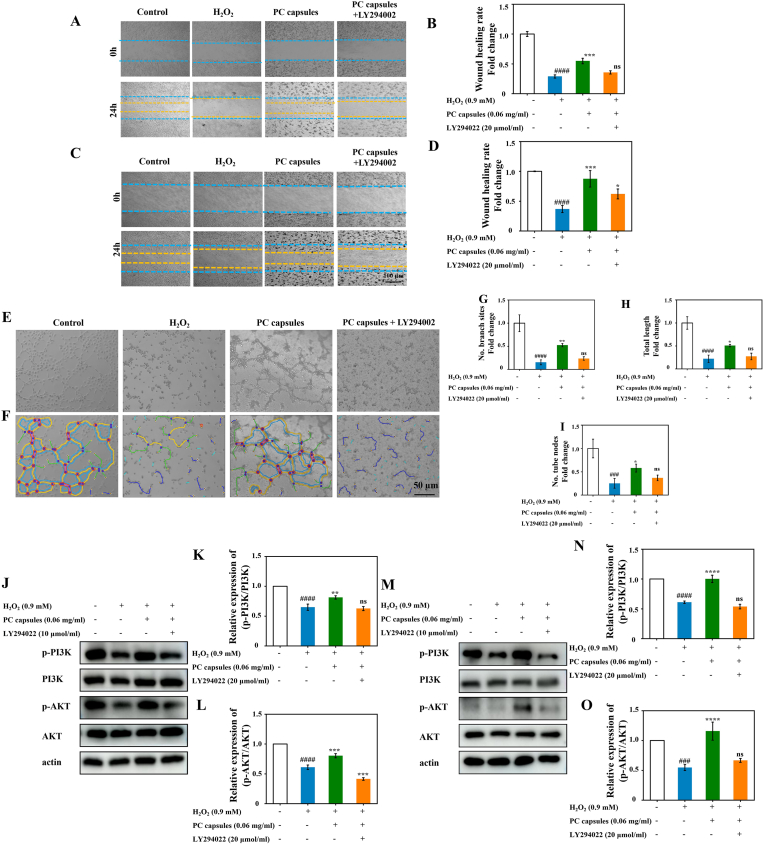
PC capsules improve HUVEC and NIH/3T3 cell dysfunction by regulating PI3K/AKT signaling. HUVECs and NIH/3T3 cells were treated with PC capsules and LY294002 for 24 h and H_2_O_2_ for 6 h. (A) Images of HUVEC migration exposure to H_2_O_2_, LY294002, PC capsules after 24 h; (B) quantification of percentage wound area remaining for HUVECs; (C) images of NIH/3T3 cell migration exposure to H_2_O_2_, LY294002, PC capsules after 24 h; (D) quantification of percentage wound area remaining for NIH/3T3 cells; (E–F) digital images of endothelial cell microtubule formation after treatment with H_2_O_2_, LY294002, and PC capsules. Quantification of (G) number of branch sites, (H) total lengths and (I) numbers of tube nodes (J); and analysis of optical density values of p-PI3K/PI3K (K) and p-AKT/AKT (L); expression of p-PI3K, PI3K, p-AKT, and AKT in NIH/3T3 cells treated with PC capsules, and LY294002 for 24 h and presence/absence of H_2_O_2_ for 6 h (M); and analysis of optical density values of p-PI3K/PI3K (N) and p-AKT/AKT (O). Data are presented as mean ± standard deviation (SD) with n = 3 independent biological replicates per group. Statistical analysis was performed using one-way ANOVA followed by Tukey's post-hoc test. Significance markers are defined as: ##p < 0.01, ###p < 0.001 (vs. control group); ∗p < 0.05, ∗∗p < 0.01, ∗∗∗p < 0.001 (vs PC group or vs LY294002 group).

The PI3K/AKT pathway plays an important role in many cellular processes such as cell migration and phenotypic transformation. To further verify the role of the PI3K/AKT pathway in the treatment of diabetic skin wounds with the PC capsules, we examined the effect of the PC capsules on the PI3K/AKT pathway in H_2_O_2_-induced cell damage in HUVECs and NIH/3T3 cells. Western blotting showed that H_2_O_2_ notably decreased the protein levels of p-PI3K and p-AKT in the HUVECs and NIH/3T3 cells. In contrast, both the PC capsules and NAC upregulated the expression of p-PI3K and p-AKT, and increased the ratios of p-PI3K/PI3K and p-AKT/AKT. Compared with the PC capsule group, no significant differences were observed in the levels of p-PI3K and p-AKT in the NAC-treated HUVECs and NIH/3T3 cells. These results further demonstrated that the PC capsules alleviated mitochondrial dysfunction and protected the HUVECs (Fig. 9A, B, C) and NIH/3T3 cells (Fig. 9D, E, F) by activating the PI3K/AKT pathway (see Fig. 10).

### 3.10 PC capsules improve HUVEC and NIH/3T3 cell dysfunction by regulating PI3K/AKT signaling

To further investigate the role of the PI3K/AKT signaling pathway in PC capsule–mediated angiogenesis, a pathway inhibition experiment was performed using the PI3K inhibitor LY294002 [33]. As shown in Fig. 10 A–D, scratch wound-healing assays demonstrated that H_2_O_2_ treatment markedly impaired cell migration in both HUVECs and NIH/3T3 cells, whereas PC capsule treatment significantly promoted migratory capacity under oxidative stress conditions. Notably, the migration-promoting effect of PC capsules was significantly attenuated upon co-treatment with LY294002. In addition, tube formation assays in HUVECs (Fig. 10 E–I) revealed that PC capsules markedly enhanced capillary-like structure formation following H_2_O_2_ stimulation, while this pro-angiogenic effect was substantially reduced in the presence of LY294002. At the molecular level, western blot analysis (Fig. 10 J–O) showed that PC capsule treatment increased the phosphorylation levels of PI3K and AKT, as evidenced by elevated p-PI3K/PI3K and p-AKT/AKT ratios. However, LY294002 significantly suppressed this phosphorylation, confirming effective inhibition of PI3K/AKT signaling [34,35]. Taken together, these results demonstrate that LY294002 suppresses PC capsule–induced angiogenesis, and that the PI3K/AKT signaling pathway plays an important role in the enhancement of angiogenesis mediated by PC capsules.

## 4 Discussion

Patients with diabetes often face significant health risks because of delayed skin-wound recovery, which represents a serious clinical challenge [36]. This biological repair mechanism involves coordinated interactions among various cellular components, particularly epidermal cells, vascular endothelial cells, and connective tissue fibroblasts [37]. The molecular mechanisms underlying poor wound healing in diabetic individuals share characteristics with other diabetes-related chronic conditions and involve multiple pathological pathways, including the activation of polyol and glucosamine metabolic routes, enhanced signaling through protein kinase C and mitogen-activated protein kinase cascades, and the accumulation of advanced glycation end-products [38,39]. Oxidative stress is a fundamental initiating factor that triggers this series of molecular disturbances [40]. Therefore, reducing oxidative stress is a critical starting point for wound healing. In our study, the ability of the PC capsules to clear H_2_O_2_, O^2−^, DPPH, and ABTS was concentration-dependent. Furthermore, the total antioxidant capacity and the ability to scavenge H_2_O_2_ and DPPH free radicals of the PC capsules were significantly better than those of Vc.

To gain a more comprehensive understanding of the antioxidant properties of the capsules, we compared the PC capsule group with a group treated with the same concentration of PC. Surprisingly, the antioxidant effect of the PC group was considerably lower than that of the PC capsule group, possibly attributable to enhanced antioxidant performance through specific structures and interactions of the PC capsules. In addition, *in vitro* experiments showed that the PC capsules reduced H_2_O_2_-mediated damage in HUVECs and NIH/3T3 cells, and enhanced cell proliferation, migration, and tube formation. We also noticed that when evaluating short-term and long-term antioxidant effects, the PC capsules not only performed well in the short term but also significantly outperformed Vc in the long-term clearance effect, which further proved their superiority in the application of antioxidants while ensuring that they provided long-term and stable antioxidant effects, which are conducive to the healing of chronic wounds in diabetes. In summary, the PC capsules exhibited significant free-radical scavenging ability and antioxidant performance, making them promising antioxidant candidates.

Recent studies showed that restoring mitochondrial homeostasis is indispensable for the effective treatment of patients with metabolic syndrome [41]. Metabolic disorders are associated with mitochondrial dysfunction and related pathological changes, and are characterized by abnormal MMP, ROS accumulation, and impaired energy synthesis [42]. Elevated glucose levels can lead to excessive ROS production and oxidative damage, which are highly sensitive in the mitochondria [43]. Therefore, we investigated the effects of the PC capsules on H_2_O_2_-induced mitochondrial dysfunction in HUVECs and NIH/3T3 cells. Consistent with the results of a previous study, H_2_O_2_ caused accumulation of mitochondrial superoxide, decreased MMP, and reduced ATP synthesis and mitochondrial mass in the HUVECs and NIH/3T3 cells. Treatment with either the PC capsules or NAC restored these parameters in the H_2_O_2_-treated HUVECs and NIH/3T3 cells. As described previously [44], mitochondrial nutrients exert protective effects through multiple mechanisms: (1) ROS scavenging and oxidative-stress mitigation [45], (2) phase 2 enzyme induction for mitochondrial repair [46], (3) enzyme cofactor provision [47], and (4) enhanced metabolic activity that promotes biogenesis [48]. In the present study, the PC capsules maintained mitochondrial homeostasis by eliminating mitochondrial ROS.

Given the remarkable therapeutic benefits of these PC capsules in wound repair, further exploration of the modulation of key signaling pathways to expedite wound closure is warranted. To elucidate the signaling mechanisms underlying the effect of the PC capsules in the treatment of diabetic skin wounds, we conducted a transcriptome analysis of skin tissues from mice receiving PC capsule therapy. An examination of the top 20 enriched pathways using the KEGG database highlighted significant enrichment of the PI3K-AKT signaling pathway. To confirm the activation of this pathway by the PC capsules, we performed IHC staining of mouse wound tissues and Western blot analysis of H_2_O_2_-treated HUVECs, NIH/3T3 cells, and chronic wound tissues. These experiments revealed decreases in p-PI3K and p-AKT expression under oxidative-stress conditions which were mitigated by PC capsule administration.

Importantly, to determine whether PI3K/AKT activation is causally involved in the pro-repair effects of PC capsules, we performed pathway inhibition experiments using the specific PI3K inhibitor LY294002. Under oxidative stress conditions, PC capsules significantly enhanced endothelial tube formation and fibroblast migration; however, these pro-angiogenic and pro-migratory effects were markedly attenuated upon co-treatment with LY294002. In parallel, LY294002 significantly suppressed the PC capsule–induced upregulation of p-PI3K and p-AKT expression. These findings indicate that the restorative effects of PC capsules on angiogenesis and cell migration are at least partially dependent on PI3K/AKT signaling activation, rather than being merely correlated with its expression changes. This pharmacological validation strengthens the mechanistic conclusion that PI3K/AKT serves as a critical regulatory hub in PC capsule–mediated functional recovery.

Studies have demonstrated that the PI3K/AKT signaling pathway plays a crucial regulatory role in wound healing, primarily through the modulation of downstream effector molecules [16]. Specifically, this pathway alleviates inflammatory responses by suppressing the expression of pro-inflammatory cytokines (such as TNF-α and IL-6), thereby creating a favorable microenvironment for wound repair [49,50]. Notably, the research team led by Wang et al. [51] identified heme oxygenase-1 (HO-1) as a key downstream target gene of the PI3K/AKT pathway. HO-1 significantly influences diabetic wound healing by regulating multiple biological processes, including inflammation, apoptosis, cell proliferation, fibrosis, and angiogenesis. Our study found that PC capsule treatment markedly reduced the expression levels of TNF-α and IL-6 in the skin tissues of diabetic mice, providing important insights into the capsules’ pro-healing mechanism. However, whether PC capsules promote wound healing by modulating other effectors of the PI3K/AKT pathway (e.g., HO-1) requires further in-depth molecular mechanistic investigation.

Recent research has indicated that PI3K/AKT serves as a primary signaling pathway in models of oxidative damage and plays a pivotal role in maintaining mitochondrial homeostasis [52]. Chen and colleagues showed that theaflavin elevated the phosphorylation and activity levels of PI3K/AKT, thereby activating nuclear factor erythroid 2-related factor 2, a critical gene involved in mitochondrial biogenesis that regulates mitochondrial quality control and promotes the elimination of ROS via antioxidant enzymes including NAD(P)H dehydrogenase quinone 1 and HO-1 [53]. This suggests that downstream effectors of PI3K/AKT are not only involved in regulating wound healing–related factors, but also play a vital role in mitochondrial oxidative stress.

Although our study demonstrates that pharmacological inhibition of PI3K significantly attenuates PC capsule–mediated cytoprotection and angiogenesis, the precise downstream effectors responsible for mitochondrial stabilization remain to be elucidated. Future studies should investigate whether additional pathways, such as Nrf2, mTOR, or HO-1 signaling, interact with PI3K/AKT to coordinate mitochondrial quality control and redox homeostasis. Moreover, genetic approaches, including siRNA knockdown or conditional knockout models, may further strengthen the mechanistic validation.

## 5 Conclusion

All in all, these self-polymerized PC capsules provided a stable, controlled, and sustained antioxidant release profile, leading to the restoration of mitochondrial homeostasis in diabetic wound-healing cells. This treatment recovered the reparative functions of fibroblasts and endothelial cells, two key wound-healing cell types, as evidenced by improved fibroblast migration and endothelial angiogenesis *in vitro*. In diabetic mice, the PC capsules significantly accelerated wound closure, enhanced collagen maturation, and lowered pro-inflammatory cytokine levels indicating an attenuated inflammatory response in the wound tissue. Mechanistically, these PC capsules introduce a novel mitochondrial repair strategy through the PI3K/Akt signaling pathway.

## CRediT authorship contribution statement

**Yifan Ping:** Conceptualization, Investigation, Visualization, Writing – original draft, Writing – review & editing. **Jiaying Wang:** Investigation, Methodology, Visualization, Writing – original draft, Writing – review & editing. **Shaoyin Wei:** Investigation, Methodology, Visualization, Writing – original draft, Writing – review & editing. **Wen Tong:** Methodology. **Wanhang Li:** Methodology. **Junxin Ren:** Investigation. **Siyi Wang:** Investigation. **Xinyi Yao:** Investigation. **Liyang Zheng:** Visualization. **Zelin Cao:** Visualization. **Dong Yang:** Conceptualization, Supervision, Visualization, Writing – review & editing. **Cuie Wen:** Supervision, Visualization, Writing – review & editing. **Shengbin Huang:** Conceptualization, Supervision, Writing – review & editing. **Shufan Zhao:** Conceptualization, Supervision, Writing – original draft, Writing – review & editing.

## Declaration of competing interest

The authors declare that they have no known competing financial interests or personal relationships that could have appeared to influence the work reported in this paper.

## Figures and Tables

**figs1:**
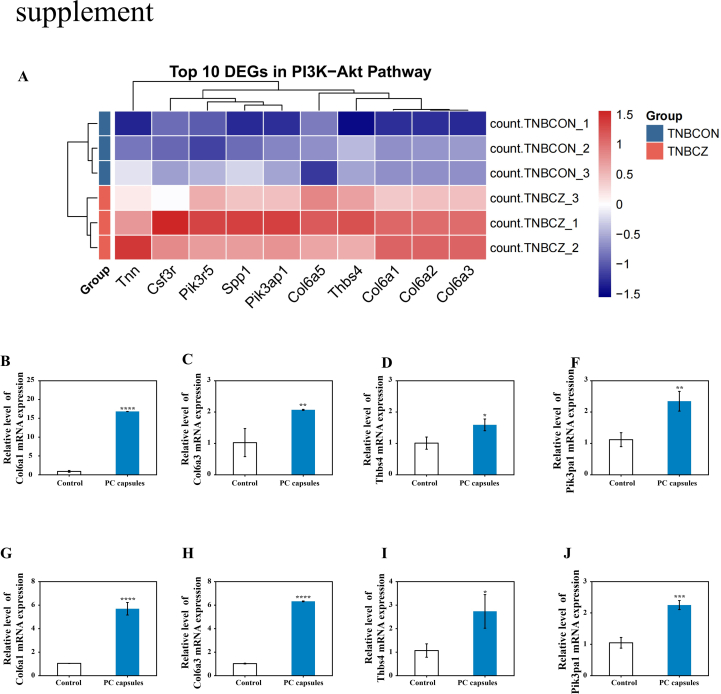
(A) Heatmap showing the top 10 differentially expressed genes (DEGs) enriched in the PI3K-Akt signaling pathway between the control group and the PC capsule treatment group. Hierarchical clustering was performed based on normalized expression levels. Red indicates relatively high expression and blue indicates relatively low expression.(B-E) RT-qPCR validation of selected key genes in NIH/3T3 cells, comparing the control group and the PC capsule treatment group.(F-I) RT-qPCR validation of the same genes in HUVECs , comparing the control group and the PC capsule treatment group.Relative mRNA expression levels were normalized to the internal reference gene and expressed as fold change relative to the control group. Data are presented as mean ± standard deviation (SD) from three independent biological replicates (n = 3 per group). Statistical analysis was performed using one-way ANOVA followed by Tukey’s post hoc test. ∗p < 0.05, ∗∗p < 0.01, ∗∗∗p < 0.001, ∗∗∗∗p < 0.0001.

## Data Availability

Data will be made available on request.
